# Atopic Dermatitis and Association of Risk for Henoch–Schönlein Purpura (IgA Vasculitis) and Renal Involvement Among Children: Results From a Population-Based Cohort Study in Taiwan

**DOI:** 10.1097/MD.0000000000002586

**Published:** 2016-01-22

**Authors:** Chang-Ching Wei, Cheng-Li Lin, Te-Chun Shen, Tsai-Chung Li, An-Chyi Chen

**Affiliations:** From the Children's Hospital (C-CW, A-CC), Management Office for Health Data (C-LL), and Division of Pulmonary and Critical Care Medicine, Department of Internal Medicine (T-CS), China Medical University Hospital, Taichung, Taiwan; and School of Medicine (C-CW, T-CS, A-CC) and Department of Public Health (C-LL, T-CL), China Medical University, Taichung, Taiwan.

## Abstract

Elevation of Th2 cytokine-driven inflammatory mediators has been reported in acute stage of Henoch–Schönlein purpura (HSP). However, the temporal interaction between Th2-mediated allergic diseases and HSP with renal involvement remains unknown. Herein, we conducted a population-based cohort analysis to investigate the risk of HSP and renal involvement in children with atopic dermatitis (AD) as 1 of the first steps in the atopic march.

From 2000 to 2007, 95,208 children with newly diagnosed AD and 190,416 randomly selected non-AD controls were included in the study. By the end of 2008, incidences of HSP in both cohorts and the AD cohort to non-AD cohort hazard ratios (HRs) and confidence intervals (CIs) were measured. Comparison of renal involvement in HSP between children with and without AD was analyzed.

The incidence of HSP during the study period was 1.75-fold greater (95% CI: 1.27–2.42) in the AD cohort than in the non-AD cohort (14.2 vs 8.11 per 100,000 person-years). The AD to non-AD HR of HSP was greater for girls (1.92, 95% CI: 1.18–3.13), children older than 6 years (2.54, 95% CI: 1.15–5.59), and those living in less urbanized area (2.74, 95% CI: 1.10–6.82). Concurrent allergic rhinitis or asthma did not increase the HR of HSP further. The HR for HSP in AD children increased from 0.67 (95% CI: 0.41–1.11) for those with ≤2 AD-related visits to 9.77 (95% CI: 6.44–14.8) for those with >4 visits (*P* < 0.0001, by the trend test). The risk of developing HSP in the AD cohort was highest within first year after AD diagnosis (HR: 3.99; 95% CI: 1.61–9.89). AD cohort with HSP had higher occurrence rate of renal involvement, particular hematuria, than non-AD cohort with HSP.

AD children had a greater risk of developing HSP and HSP with renal involvement. Further research is needed to clarify the role of allergy in the pathogenesis of HSP and renal involvement.

## INTRODUCTION

Henoch–Schönlein purpura (HSP), nowadays called IgA vasculitis, is an immune complex vasculitis affecting small vessels with dominant IgA deposits.^1^ HSP is a systemic vasculitis commonly seen in children.^1^ HSP is characterized by nonthrombocytopenic purpura, abdominal pain and bleeding, arthritis, and renal involvement.^1^ The manifestations of renal involvement vary from asymptomatic hematuria and/or proteinuria to nephritic syndrome, nephrotic syndrome, and rapidly progressive glomerulonephritis.^2^ The long-term outcome of HSP depends primarily on the extent of renal manifestations.^2–4^

HSP had been termed anaphylactoid purpura because HSP was reported to develop after exposure to allergens in drugs or food, immunization, and insect bites.^5–8^ Although the exact etiology and pathogenesis of HSP remain unclear, elevated serum IgA levels, vascular deposition of IgA-containing immune complexes, and increased proinflammatory cytokine levels were observed during its acute stage.^9^ Moreover, previous studies revealed elevated soluble IL-2 receptor levels and a shift toward increased Th1 lymphocyte production in children with HSP, indicating a Th1 polarized disease.^10,11^ However, Davin et al^12^ reported that plasma IgE levels were significantly higher in patients with HSP, indicating a shift toward Th2 immune response. The complexity of Th1/Th2 interaction of HSP raises an research interest whether Th2-mediated allergic diseases have any influence on the development of HSP and HSP with renal involvement.

Atopic dermatitis (AD) is a Th2-driven chronic relapsing inflammatory skin disease.^13^ AD is classically the first clinical manifestation of allergic disease, presenting early in infancy, followed by the development of allergic airway diseases in some children.^13–15^ This so-called atopic march suggests a common etiology for the different atopic diseases.^15–17^ The peak age of HSP occurrence is 4 to 6 years. Hence, AD children are an appropriate cohort to assess the temporal interaction between allergy and HSP. We conducted a population-based cohort study to investigate the incidence and subsequent risk of HSP and HSP with renal involvement in Taiwan children with AD so that we could provide better insights into the common immunological aberrancies of both disorders. We hypothesized AD children had higher risk of HSP and HSP with renal involvement.

## METHODS

### Data Sources

The National Health Insurance Research database (NHIRD), maintained by the National Health Research Institutes, contains population-based claims data from the National Health Insurance program, a mandatory-enrollment, single-payment system created in 1995, now covering over 99% of Taiwan's population (http://www.nhi.gov.tw/english/index.aspx).^18^ The high reliability of the diagnostic data from the NHIRD has been evaluated in previous studies.^19–22^ This study utilized a data file derived from the NHIRD, containing information from half of all insured children in Taiwan, chosen at random. The dataset provided a sufficient sample size to pursue the objectives addressed in this study. Because of personal electronic data-privacy regulations, identifying information was encrypted before being sent to the researcher. The study was approved the Institutional Review Board of China Medical University Hospital (CMUH104-REC2-115). Diseases were coded based on the International Classification of Diseases, 9th Revision, Clinical Modification (ICD-9-CM).

### Study Design and Subjects

**FIGURE 1:**
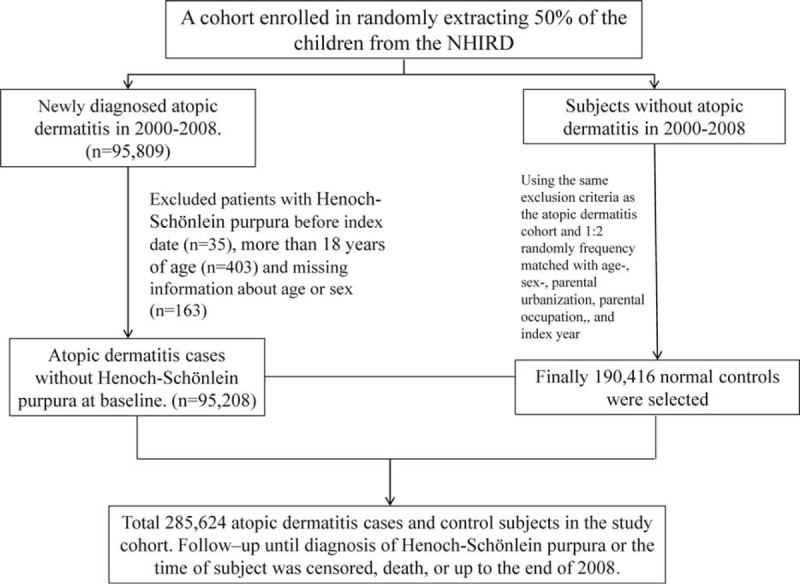
Flow diagram of participant selection.

AD is a chronic, relapsing, inflammatory skin disorder. Hence, AD was defined as at least 1 inpatient claim record or 2 ambulatory claims containing ICD-9-CM code 691.8 in the primary diagnosis field. A total of 95,208 patients (ages <18 years) newly diagnosed with AD between 2000 and 2007 were identified as the AD cohort. The baseline index date was the date of AD diagnosis. For each child with AD, we randomly selected 2 children without AD (never having ICD-9-CM code 691.8 in any diagnosis field) matched by sex, age (within 1 year), urbanized residence area, parental occupation, and baseline year. We used the method of 1:2 matching to increase statistical power and to control for potential confounders. Children with missing data regarding sex or date of birth and those with preexisting HSP (ICD-9-CM 287.0) before or within 1 month after the baseline index date were excluded. We also identified and compared the differences of the subjects who were diagnosed with allergic rhinitis (ICD-9 code: 477) and asthma (ICD-9 code: 493) in both the AD and non-AD cohorts. Diagnoses of HSP were based on clinical manifestations, including nonthrombocytopenic purpura located predominantly on dependent areas, such as the lower extremities and buttocks, and at least 1 of the following findings: arthralgia or arthritis, abdominal pain, or nephritis, according to the 1990 criteria of the American College of Rheumatology (ACR). In this study, IgA vasculitis and allergic diseases were diagnosed by physicians and were defined as at least 1 inpatient claim record or 2 ambulatory claims in any diagnosis field with the respective ICD-9-CM codes. IgA vasculitis with renal involvement was defined as occurrence of any 1 of the following: hematuria, proteinuria, nephritis, and nephrotic syndrome (ICD-9 codes 599.7, 791.0, and 580–585) within 1 year after IgA vasculitis was diagnosed. Each child was followed from the index date until the development of IgA vasculitis, withdrawal of insurance, or conclusion of follow-up on December 31, 2008. This study's design is summarized in the flow chart shown in Figure 1.

### Statistical Analysis

The sociodemographic variables included in this study were sex, age, urbanization, and parental occupation. Urbanization was categorized into 4 levels based on the population density of the residential area: level 1 was the most urbanized and level 4 was the least urbanized. All data analyses were performed using SAS software version 9.1 (SAS Institute, Inc., Carey, NC). Statistical significance was set at *P* < 0.05 in 2-tailed tests. To describe the baseline distributions of the AD and non-AD cohorts, mean and standard deviations (SDs) were used for continuous variables and counts and percentages were used for categorical variables.

Differences were examined using the chi-squared test for categorical variables and *t* test for continuous variables. Kaplan–Meier method was used to estimate the proportion of study subjects in both cohorts who did not develop IgA vasculitis during the follow-up period. The incidence was calculated for each cohort. Hazard ratios (HRs) and 95% confidence intervals (CIs) were calculated using multivariate Cox proportional hazard regression models, with the non-AD cohort as the reference group, to assess the association between AD and the risk of developing IgA vasculitis.

## RESULTS

**FIGURE 2:**
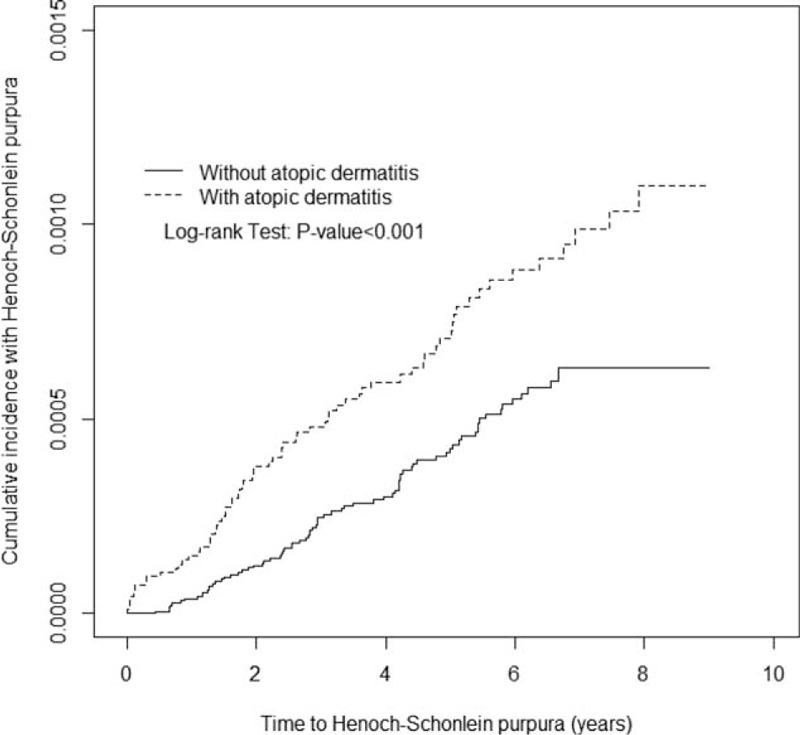
Cummulative incidence of Henoch-Schönlein purpura for patients with atopic dermatitis (dashed line) or without atopic dermatitis (solid line).

This study evaluated 95,208 AD cases and 190,416 non-AD cases. No differences in the sociodemographic variables were noted between groups. Majority of AD children were boy (54.1%), ages ≤2 years (62.3%), and lived in higher urbanization regions (33.8%; Table 1). AD cohort had higher occurrence rate of allergic rhinitis (18.5% vs 4.92%) and asthma (11.4% vs 2.64%) than no-AD cohort (Table 1). There were no differences in age group, sex, urbanization of living areas, or follow-up period between the 2 cohorts. Kaplan–Meier analysis showed that the accumulated incidence rate of HSP was significantly greater in the AD cohort than in the non-AD cohort (log-rank test, *P* < 0.0001; Figure 2).

**TABLE 1:**
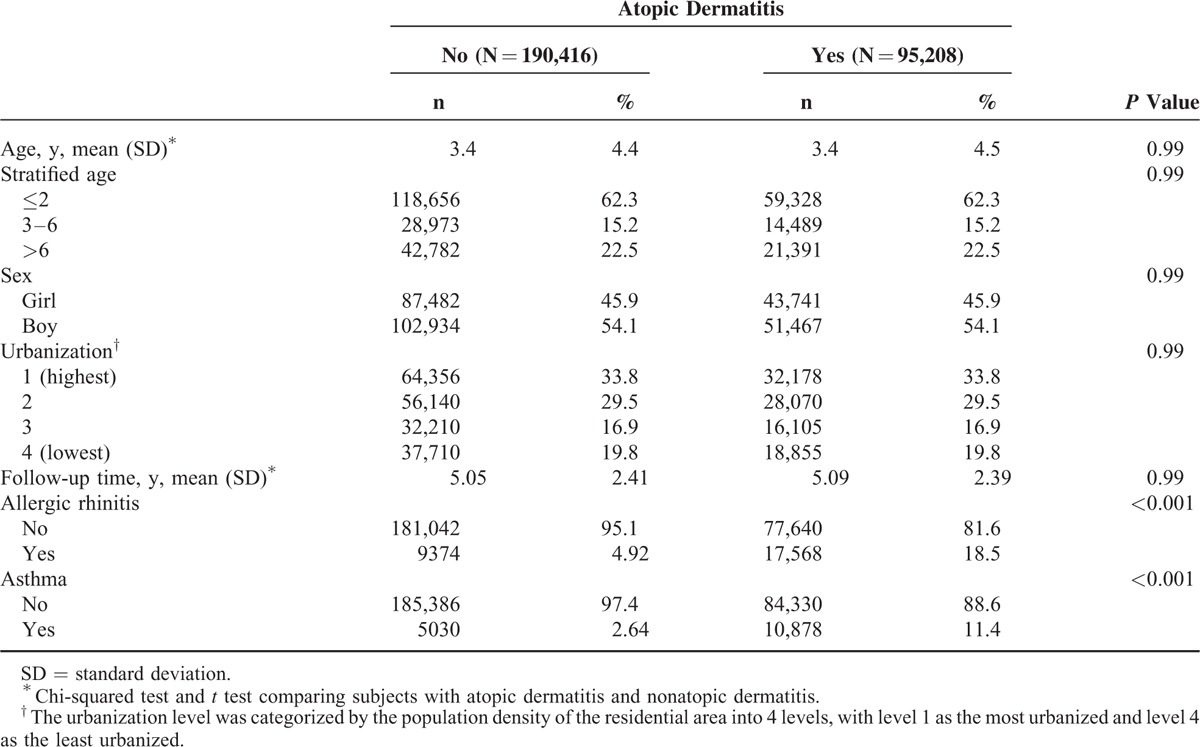
Demographics Between Children With and Without Atopic Dermatitis

The incidence of HSP in both cohorts and the AD to non-AD HRs of HSP by sociodemographic status are presented in Table 2. Overall, the HSP incidence was 1.75-fold greater (95% CI: 1.27–2.42) in the AD cohort than in the non-AD cohort (14.2 vs 8.11 per 100,000 person-years) at the end of follow-up period. The increased AD to non-AD HRs of HSP was consistent regardless of age, sex, and urbanization. The AD to non-AD HRs were increasing with age, from 1.65 for HSP (95% CI: 1.08–2.52) for the children ages <2 years to 2.54 (95% CI: 1.15–2.51) for the children ages >6 years. The sex-specific AD to non-AD HRs was greater for girls 1.92 (95% CI: 1.18–3.13) than for boys 1.63 (95% CI: 1.05–2.51). The urbanization-specific AD to non-AD HRs decreased with the higher level of urbanization. Concurrent allergic rhinitis or asthma did not increase the risk of HSP further in AD cohort (Table 2). The HR for HSP in AD children increased from 0.67 (95% CI: 0.41–1.11) for those with ≤2 AD-related visits to 9.77 (95% CI: 6.44–14.8) for those with >4 visits (*P* < 0.0001, by the trend test) and the trend was consistent in both sex (Table 3). The risk of developing HSP in the AD cohort was highest within first year after AD diagnosis (HR: 3.99; 95% CI: 1.61–9.89) and declined with follow-up time (Table 4). AD cohort with HSP had higher occurrence rate of renal involvement, particular hematuria (*P* = 0.04), than non-AD cohort with HSP (Table 5).

**TABLE 2:**
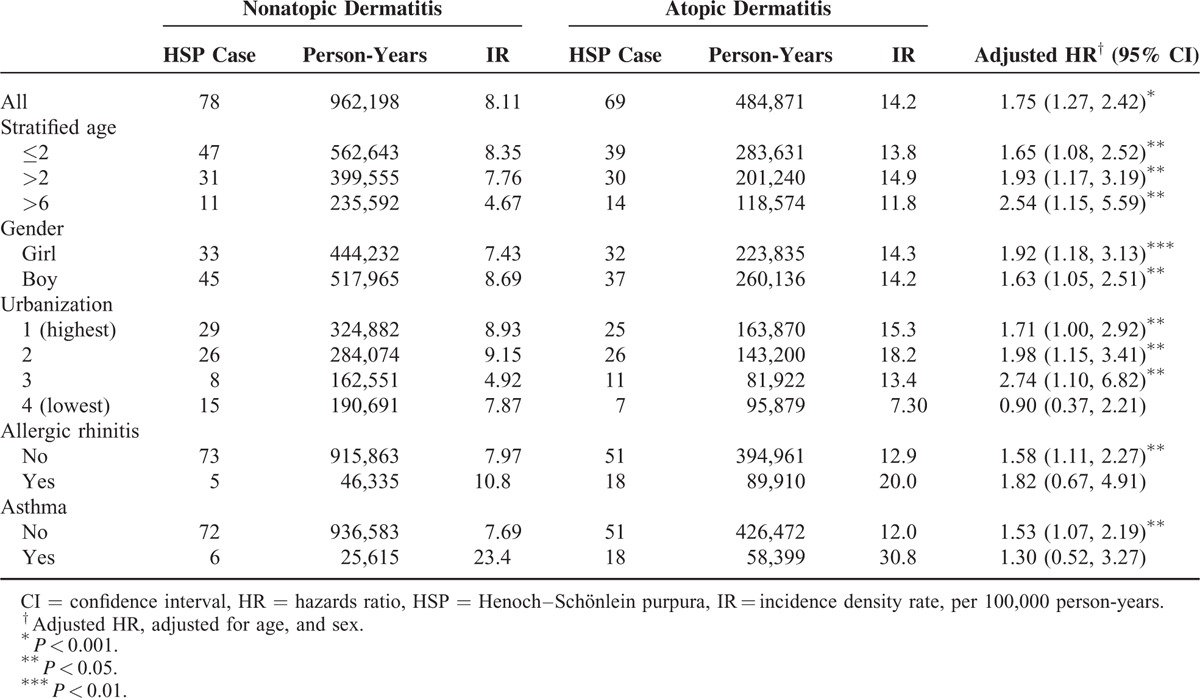
Incidence and Relative Risk of Henoch–Schönlein Purpura in Children With Compared to Children Without Atopic Dermatitis Stratified by Demographics in Cox Proportional Hazard Regression

**TABLE 3:**
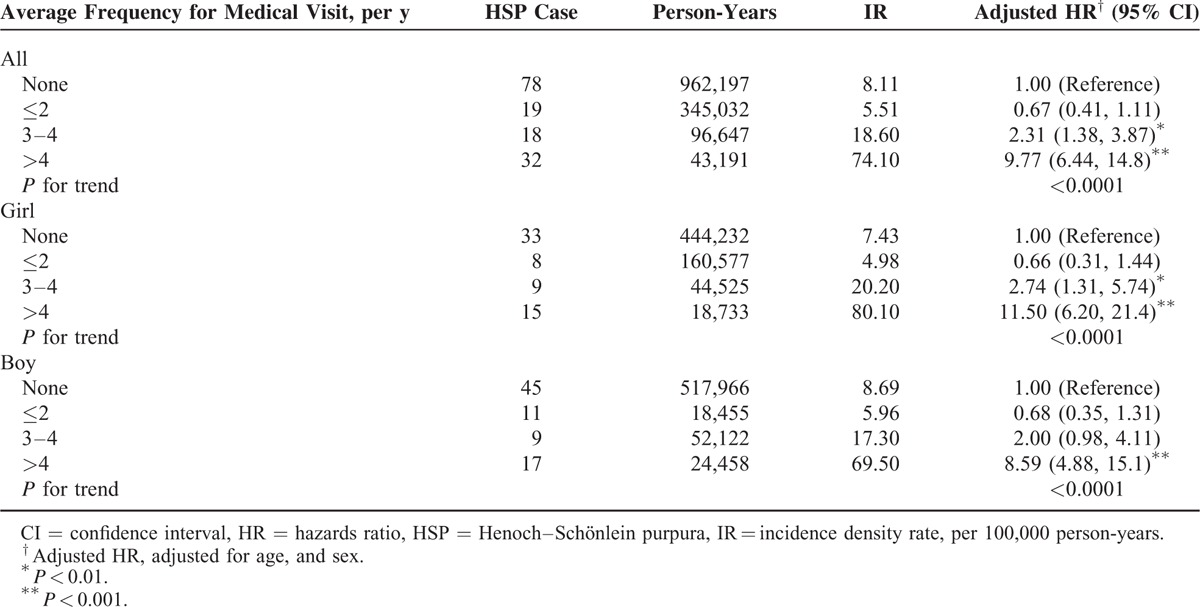
Incidence and Relative Risk of Henoch–Schönlein Purpura in Children Stratified by Annual Frequency of Medical Visits for Atopic Dermatitis in Cox Proportional Hazard Regression

**TABLE 4:**
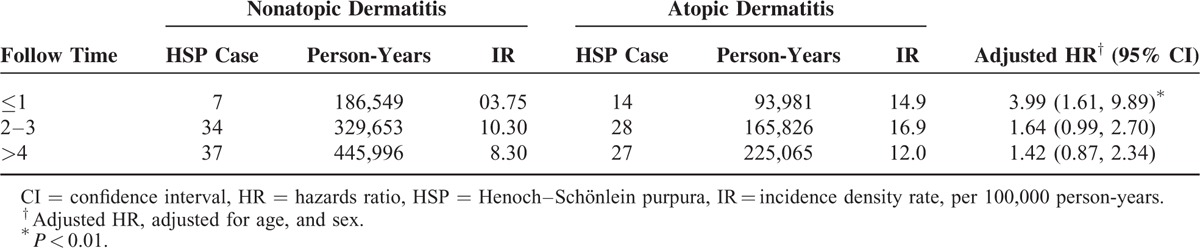
Incidence and Risk of Henoch–Schönlein Purpura in Children With Atopic Dermatitis Compared to Children Without Atopic Dermatitis, Stratified by Follow-Up Years

**TABLE 5:**
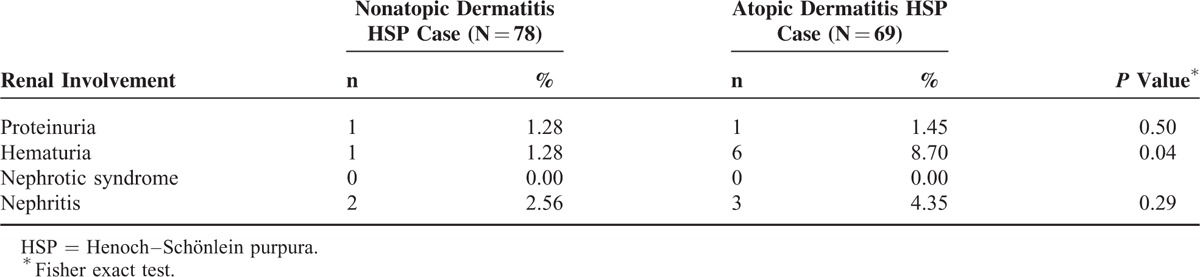
Comparison of Renal Involvement in Henoch–Schönlein Purpura Between Children With Atopic Dermatitis and Without Atopic Dermatitis

## DISCUSSION

This is the first population-based cohort study investigating the incidence and risk of HSP and HSP with renal involvement in AD cohort compared with non-AD cohort. Our results showed that AD was a risk factor for HSP and HSP with renal involvement in children. The risks for HSP were constantly increased regardless of age, sex, and urbanization of residential area. The elevated risk of HSP was greater for girls, older children, and those living in less urbanized area.

Previous epidemiological studies revealed the annual incidence of HSP 13.5 to 18/100,000 children with slight male predominance and peak age between 4 and 6 years.^1,24,25^ The present study demonstrated the incidence rate of HSP 8.1/100,000 person-years in non-AD children and 14.2/100,000 person-years in AD children in Taiwan between 2000 and 2008. In addition, boys had a higher incidence in non-AD cohort and had a slightly lower incidence than girls in AD cohort, indicating that the impact of AD is greater for girls than for boys. The incidence of HSP is generally inversely associated with age in both AD and non-AD cohorts. However, the risk of HSP in AD to non-AD cohort increased as children grew. The discrepancy between sexes and age groups was not well documented in previous studies.^1,24,25^

Concurrent allergic rhinitis or asthma seems not related to a further increase of risk for HSP. The risk of HSP increased with more clinical burden of AD and was highest within first year after AD was diagnosed. AD cohort with HSP had higher occurrence rate of renal involvement, particular hematuria, than non-AD cohort with HSP. Although most of the children with HSP have a good outcome, long-term prognosis of HSP depends on the severity of renal involvement. Previous studies reported that severe abdominal symptoms, older age, and persistent purpura, decreased factor XIII activity increased the risk of renal involvement.^26,27^ However, no any study except ours investigated whether AD increased risk of HSP with renal involvement. Our data indicate that AD is a risk factor for HSP and HSP with renal involvement.

AD is a chronic inflammatory skin disorder that develops in early infancy, often preceding asthma and allergic disorders, and representing the beginning of the atopic march.^15^ Therefore, the AD cohort is a useful model to study the impact of atopy on certain diseases. In this study, 62.3% of children were diagnosed with AD before age 2, and boys had a slightly higher AD prevalence. This age and sex distribution is similar to previous reports in Asian populations.^25^ Recent advances in our knowledge of the pathogenesis of AD have posited the relationship between immune dysregulation, and skin barrier abnormalities.^13,14^ Exposure to irritants and allergens from barrier-defective skin in patients with AD may lead to a specific Th2 cell activation and IgE production in a subset of these patients. Elevated serum IgE is also commonly found in children with HSP.^12^ The genetic predisposition of an individual to make IgE antibody responses to common environmental allergens and triggers may be associated with the development of AD and IgA vasculitis.

To date, the exact triggers and pathogenesis of HSP remain unclear. HSP was originally considered a Th1-mediated systemic vasculitis with cytokine cascades and endothelial cell lesions in small vessels in the acute stage.^9,11,29^ Th1-related serum cytokines such as IL-1, IL-6, and tumor necrotic factor levels and soluble IL-2 receptor expressions are often elevated in the acute phase of HSP.^9,11,29^ In contrast, there have also been several reports of elevated Th2-related biomarker levels in children with HSP.^10,12,30,31^ Allergic diseases are caused by inappropriate Th2 cell-mediated immunological responses to allergens. Th2 cells secrete IL-4 and IL-5, which lead to type 1 hypersensitivity.^29^ The onset of HSP usually follows viral infection, bacterial infection, drug allergy, food allergy, insect bites, or immunization, which implies that allergic reactions may participate in the onset of this disease.^5–8^ In the presence of IL-4, the majority of B-cells switch immunoglobulin production to produce a vast amount of IgE.^30,31^ Davin et al^12^ reported that the incidence of increased plasma IgE levels was significantly higher in patients with HSP and suggested that IgE plays a possible pathogenic role in HSP. IL-5 switches the B-cell immunoglobulin to IgA and activates eosinophils.^30,31^ IgA is considered to be the mediating factor in IgA vasculitis.^1^ Namgoong et al^10^ found that eosinophil cationic protein (ECP) and soluble IL-2 receptor levels were significantly higher in patients with IgA vasculitis than in a control group, indicating that T-cell and eosinophil activation were involved in the pathogenesis of IgA vasculitis. Tsuji et al found higher serum IgE and urinary leukotriene E4 levels at the onset of IgA vasculitis in children with AD than in healthy children.^30^ They also noted IgE deposits in the Langerhans and mast cells in patients with IgA vasculitis with renal involvement, implying that stimulation of IgE-sensitized mast cells by specific antigens in the presence of circulating IgA immune complexes was associated with the disease cascade.^27^ In turn, the increased capillary permeability and perivascular deposition of IgA CIC, which resulted in deposition of IgA and complement C3 in the small vessels of the skin and renal glomeruli.^10,28^ Kawasaki et al found elevated serum IL-4 levels in patients with acute IgA vasculitis without renal involvement and elevated IL-5 and ECP-activated eosinophil levels in patients with IgA vasculitis with renal involvement.^32^ These studies support our findings. More recently, Li et al^16^ reported significantly elevated Th2 and Th17 cell levels in patients with acute IgA vasculitis. Dysregulation of the immune response, such as functional imbalance of Th cell subsets and a strongly polarized cytokine milieu in the peripheral blood, to environmental stimuli may contribute to both allergic and autoimmune diseases.^16^ Therefore, both early-life environmental factors and common immune aberrancy may contribute to the development of allergies and IgA vasculitis.

This study had several limitations. Thorough medical records for each patient are not available from the NHIRD. Therefore, the clinical presentations such as arthritis, bowel angina, complications, and therapeutic strategies in these patients and the clinical information of AD, such as serum IgE level, eosinophil level, skin infection, a family history of allergic diseases were unknown. Another limitation was the lack of data on genetic and environmental factors that might affect the risks of HSP and allergic diseases. In this study, diagnoses of HSP were made by physicians were based on the criteria defined by the 1990 criteria of ACR. The reliability of HSP diagnosis according to the ACR criteria was reported a sensitivity of 87.1% and a specificity of 87.7%.^23^ Our results showed an incidence of 14.2 versus 8.1 per 100,000 person-years of HSP in the AD versus the non-AD population. Hence, the result of the 1.75-fold greater risk of HSP in the AD cohort versus the non-AD cohort has a quite low clinical relevance taking account to the low prevalence of HSP. Lastly, the ethnic population in the study was Chinese, which may not yield results generalizable to other populations.

In conclusion, this population-based cohort study reveals an increased incidence and subsequent risk of IgA vasculitis in children with AD. Future studies to clarify the role of allergy in the pathogenesis of IgA vasculitis with or without renal involvement are recommended.
